# Vertical stratification of the water microbiome in an electric water heater tank: implications for premise plumbing opportunistic pathogens

**DOI:** 10.2166/wh.2024.265

**Published:** 2024-11-21

**Authors:** Vicente Gomez-Alvarez, Hodon Ryu, Morgan McNeely, Christy Muhlen, Daniel Williams, Darren Lytle, Laura Boczek

**Affiliations:** US Environmental Protection Agency, Office of Research and Development, Cincinnati, OH 45268, USA

**Keywords:** hot water tank, Legionella pneumophila, microbial communities, opportunistic pathogens, premise plumbing, temperature stratification

## Abstract

Hot water systems are the most frequent environment associated with the prevalence and growth of opportunistic premise plumbing pathogens (OPPPs). Previous studies identified water heaters as a source of waterborne diseases and concluded that design variables may contribute to their prevalence. A multifaceted approach was used to investigate the vertical stratification of the microbiome and selected OPPPs in an electric water heater tank connected to a home plumbing system simulator. Results show that the microbiome is highly diverse with evidence of temperature stratification and temporal structuring influenced by the partial drainage of the tank. Representatives of the *Mycobacterium* spp. were the most prevalent taxa, followed by *Legionella* spp., and a relatively low prevalence of *free-living amoeba Vermamoeba vermiformis*. Higher concentrations of *Legionella pneumophila* at the bottom of the tank indicated the potential growth and protection of this opportunistic pathogen at this location. Overall, partial drainage of the water tank (60% of the tank capacity) did not significantly mitigate the microbiome and selected OPPPs. The outcome of this study sheds light on the role of vertical stratification on water quality and demonstrates the resilience of the microbial community residing in an electric water heater tank and the implications for public health.

## GRAPHICAL ABSTRACT

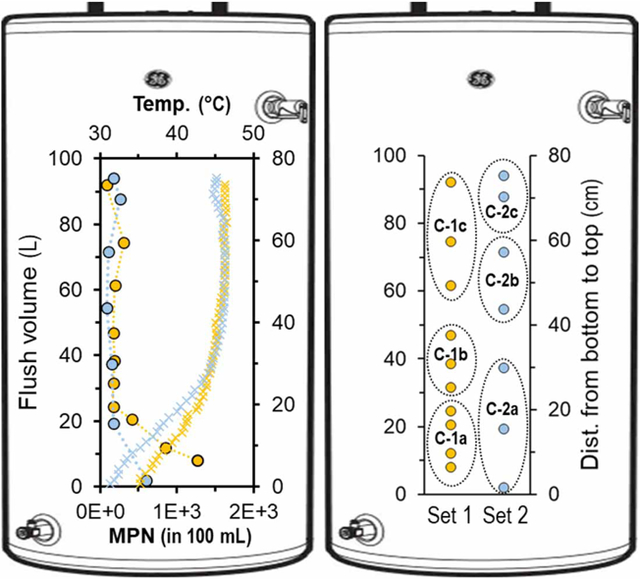

## 1. INTRODUCTION

Public health data show that a significant fraction of the nation*’*s waterborne disease outbreaks are associated with premise plumbing in built environments. Surveillance data in the United States (US) indicate an increasing trend in the annual proportion of reported outbreaks associated with premise plumbing deficiencies (Craun *et al.* 2010). Premise plumbing includes the portion of the drinking water distribution system that connects the main via the service line to public and private houses and occupational buildings, and the hot and cold water plumbing within the building (NRC 2006). Water quality within the premise plumbing is not monitored by the US Environmental Protection Agency (EPA) regulations, except for the Lead and Copper Rule (LCR). Despite the implementation of disinfection strategies, studies in homes and occupational buildings have suggested that the unique characteristics of premise plumbing (e.g., large surface area to volume ratio, long water age, favorable temperature, and low or no disinfectant residual) provide a favorable environment for microbial proliferation including the growth of pathogenic bacteria that cause infectious disease (NRC 2006).

Opportunistic premise plumbing pathogens (OPPPs) previously identified include *Legionella pneumophila* and nontuberculous mycobacterial (NTM) species such as *Mycobacterium avium*, *M. intracellulare*, and *M. abscessus*. *L. pneumophila* is the primary causative agent of Legionnaires*’* disease (Phin *et al.* 2014) and can colonize and persist within drinking water systems (Berjeaud *et al.* 2016). NTM species are increasingly recognized as important opportunistic pathogens of humans, and data indicates a global increase of these agents in reports of infection and disease (Dahl *et al.* 2022). Representatives of the *M. avium-intracellulare* and *M. abscessus* complexes are considered an emerging worldwide public health threat and one of the leading causes of nosocomial infections (Moradali *et al.* 2017).

Hot water premise plumbing systems are the most frequent environment associated with the prevalence of OPPPs (Falkinham *et al.* 2015), and there is evidence that water heater tanks allow these organisms an opportunity to proliferate (Gomez-Alvarez *et al.* 2023; Song *et al.* 2023). Typically, a hot water device is used to heat the water and store water. This device may have a built-in gas burner system or an electric heating element. Previous studies implicate electric water heaters with a higher prevalence of *Legionella* colonization than gas heater devices (Alary & Joly 1991; Dufresne *et al.* 2012). Further studies concluded that design variables, water column stratification, and sedimentation also contribute to *Legionella* colonization in electric water heaters (Brazeau & Edwards 2013a; Rhoads *et al.* 2020). Stratification in aquatic ecosystems creates vertical isolation of portions of the water body with the effect of clustering the microbiome through the water column. The effect on the water microbiome, including OPPPs that might be associated with the column stratification in electric water heater tanks, has not been extensively studied. Therefore, the objectives of this study were to characterize the microbial community and to examine how populations of OPPPs, along with free-living amoeba (FLA), are influenced by the vertical stratification in an electric water heater tank connected to a simulated home plumbing system (HPS). Specifically, water samples were sequentially collected from the water tank and analyzed for occurrence of *Legionella* spp. and *Mycobacterium* spp. along with the FLA *Vermamoeba vermiformis* using quantitative PCR (qPCR). *V. vermiformis* is a waterborne FLA with very low pathogenicity that can act as a host for *L. pneumophila*, *Pseudomonas aeruginosa*, and several NTMs (Delafont *et al.* 2018). Furthermore, a 16S rRNA gene sequencing approach was used to investigate the vertical stratification of the microbial community and their response to a partial drainage of the hot water tank (i.e., sequential sampling). The outcome of this study will shed light on the role of vertical stratification on microbial water quality in electric hot water tanks and implications for OPPPs and public health.

## 2. MATERIALS AND METHODS

### 2.1. HPS simulator

The HPS simulator was constructed in the EPA*’*s Andrew W. Breidenbach Environmental Research Center (AWBERC) (Cincinnati, Ohio) and has been continuously operated since 2012 (Lytle *et al.* 2021). The simulated HPS includes copper pipes, a 40-gallon (≈151 L) electric 2-element hot water tank (model GE40M06AAG), a dishwasher, a washing machine, bathroom sinks, a shower, and a toilet (Supplementary Figure S1). The cold water lines and the hot water tank are fed with water that is supplied by the building water supply (chlorinated drinking water). The flush protocol (i.e., daily use by a four-person residence) consisted of manually opening and running the faucets, shower, and toilet for a combined volume of 70–80 gallons per day. Hydraulic retention time (HRT) defined as the number of hours the water remains in the water tank during the flush protocol is 13.6 to 11.9 h. Detailed information on the design and flushing protocol for the HPS was described previously (Gomez-Alvarez *et al.* 2023) and can be found in Supplementary Materials and Methods.

### 2.2. Sequential sampling

**Figure 1 |:**
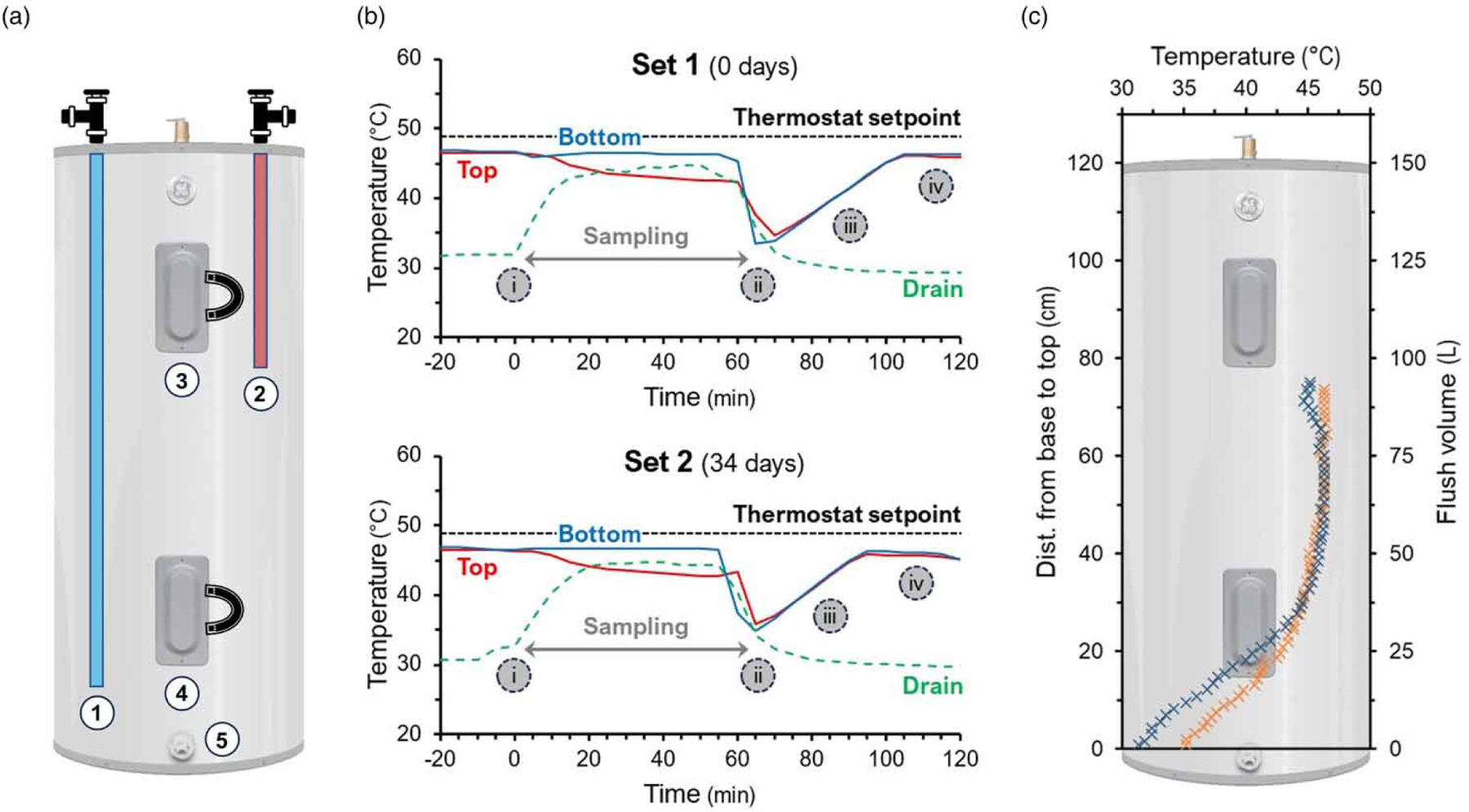
(a) Schematic of the electric water heater tank (model GE40M06AAG) with components enumerated from 1 to 5. (b) Temperature profiles obtained from temperature sensors attached to the upper element (Top, <inline image>) and the lower element (Bottom, <inline image>) of the tank, and from water samples obtained from the drain valve (Drain,<inline image>) located at the base of the tank (see component 5 in (a)). The sequential sampling profile (i.e., steps) is described as follows: (i) turn off thermostat and cold water valve off, followed by first flushing/sample collection, (ii) last flushing/sample collection followed by cold water valve on and turn on of the thermostat, (iii) heating elements and water temperature rise, and (iv) reach initial setpoint of water temperature (——). (c) Water samples and temperature were collected over the course of two sets of sampling events from the drain valve. Legend: Set 1 (<inline image>); Set 2 (<inline image>).

Hot water samples were obtained from a sampling port (drain valve) located at the base of the hot water tank (Figure 1(a) component 5). A1-h sampling procedure (flushing and collection of water samples) was designed to mitigate the effect of drainage of the tank on the HPS operation (Figure 1(b)). The HRT during the sampling procedure was 1.7 h. The sequential sample volumes outlined in this study were collected after turning off the thermostat and closing the cold water valve to prevent the disturbance of the incoming cold water to the existing water tank system. After the last flushing/sample collection, the cold water valve and the thermostat were turned on. The heating elements and water temperature thus equilibrated to their initial setpoint. Two sets (Sets 1 and 2) of samples were obtained following a sequential sampling protocol, meaning that 1 L of water was collected for analysis (e.g., microbial) after a predetermined volume of water was flushed from the water tank (Table 1). Set 2 (July 2023) was collected 34 days after Set 1 (June 2023). A previous study on this system indicates that the microbial population within the HPS is sensitive to residential activities (i.e., disturbances) and has the capacity recover to their original state after a period of 30 days (Gomez-Alvarez *et al.* 2023). Sequential samplings resulted in a partial drainage of the hot water tank (90 L of flushed water is equivalent to 60% of the tank capacity). The water temperatures of flushed and collected water samples were measured immediately after collection with a handheld thermometer (Fisher Scientific, Hampton, NH). Additional temperature recordings were obtained from temperature sensors attached to the top, bottom, and drain in the water tank and collected using a HOBO UX120–006M 4-channel analog data logger (Onset, Bourne, MA). Sample T01 (initial flush) for both sets contained a portion of stagnated water from the drain valve/port and was discarded prior to further analysis.

### 2.3. DNA extraction, 16S rRNA gene sequencing, and reads processing

The biomass of each water sample (approximately 1 L) was immediately concentrated via filtration using 0.2-μm sterile membranes (Pall Corporation, Port Washington, NY), and subsequent total DNA extractions were performed according to the manufacturer*’*s protocol (DNeasy PowerWater kit, Qiagen). DNA concentrations were measured using a NanoDrop ND-1000 UV spectrophotometer (NanoDrop Technologies, Wilmington, DE). DNA extracts were stored at –20 °C until further processing. The V4 region of the 16S rRNA sequence was amplified using the bacterial primer set 515F and 806R (Caporaso *et al.* 2012). Paired-end 250 bp reads were generated using the MiSeq® platform (Illumina Inc., San Diego, USA) and screened following the procedure described in Gomez-Alvarez *et al.* (2016). Detailed information on PCR amplification, sequencing, and processing of reads can be found in Supplementary Materials and Methods. After quality control filtering and removal of artificial sequences, 657,571 reads were retained from 16S rRNA bacteria libraries.

### 2.4. Microbial community assemblages

Prior to community analysis, 16S rRNA gene libraries were rarefied to the smallest data set of 17,500 bacteria reads. Bacteria analysis identified 395 operational taxonomic units (OTUs). Normalized libraries were used to calculate the richness estimator (ChaoI), Shannon diversity (*H* ), and the square root of the Jensen–Shannon divergence matrix that describes the dissimilarity (1-similarity) among multiple groups. ChaoI is an estimator of species richness which corrects for species that might be present but not detected in the sample due to low abundance and *H* measures the diversity of species in a community. The Jensen–Shannon divergence is a method of measuring the similarity between two probability distributions based on relative abundance. Alpha diversity indexes (ChaoI and *H* ) and the distance matrix were generated with the software mothur v1.48.0 (Schloss *et al.* 2009). Taxonomy was assigned using the SBDI Sativa curated 16S GTDB r207 reference database (SBDI 2021). Detailed information on taxonomic classification can be found in Supplementary Materials and Methods.

### 2.5. Multivariable ordination and statistical analysis

Nonmetric multidimensional scaling (nMDS) and cluster analysis (CA) were used to describe the relationships among microbial communities. For nMDS, the stress score was used to interpret the goodness-of-fit of the regression of the observed distances among the samples (Clarke 1993). The stress score is a measure of how well the ordination fits the data with values of <0.05, excellent; <0.1, good; <0.2 weak; and ≥0.2 random representation in reduced dimensions. The nMDS and CA were based on the square root Jensen–Shannon divergence (dissimilarity) matrix. A one-way permutational multivariate analysis of variance (PERMANOVA) test was applied on the distance matrix with 9,999 permutations to determine if there were significant differences (α = 0.05) between the microbial communities (Anderson 2001). Similarity Percentage (SIMPER) analysis was conducted to determine the percentage contribution of species to the differences observed within water sections (Clarke 1993). Differentially abundant OTUs were identified using the linear discriminative analysis (LDA) effect size (LEfSe) with an LDA threshold score = 2.0 and α = 0.05 implemented in the software mothur. LEfSe determines the OTUs (i.e., features) most likely to explain differences between microbial communities (i.e., clusters) by coupling standard tests for statistical significance with biological consistency and effect relevance (Segata *et al.* 2011). A Mann–Whitney *U* test (α = 0.05) was used to evaluate the differences in alpha diversity indexes and taxa abundance. Pearson*’*s correlation coefficient (*r*) was calculated for each pairwise combination of variables (e.g., OPPP abundance vs. temperature) in the samples analyzed. The significance of the correlation test was set at 95% ( *p* ≤ 0.05). Ordination plots and statistical analysis were performed with the software PAST v4.14 (Hammer *et al.* 2001). A Newick-formatted dendrogram comparing communities was constructed using the software MEGA 11 v11.0.13 (Tamura *et al.* 2021).

### 2.6. Quantitative polymerase chain reaction (qPCR)

Culture-independent qPCR analyses were conducted for the genera *Legionella* and *Mycobacterium* along with the FLA *V. vermiformis*. In addition, qPCR was used to monitor the occurrence of two major OPPP species: *L. pneumophila* and *Mycobacterium intracellulare*. All qPCR assays were performed using a QuantStudio™ 6 Flex system (Applied Biosystems, Foster City, CA) following the procedure described in Ryu *et al.* (2013). Detailed information on probes, gene targets, and amplification protocols from the purified DNA can be found in Supplementary Table S1.

### 2.7. Culturable *L. pneumophila*

The presence and quantification of culturable *L. pneumophila* was determined using the 10 mL potable water Legiolert assay as outlined by the manufacturer (IDEXX, Westbrook, Maine). Detailed information on the Legiolert assay can be found in Supplementary Table S1.

## 3. RESULTS AND DISCUSSION

### 3.1. Vertical stratification in an electric water heater tank

A sequential sampling protocol was implemented to establish a vertical water profile of an electric 2-element heater tank (Figure 1(a)) connected to an HPS simulator. Two sets (Sets 1 and 2) of samples were collected for this study, with Set 2 collected 34 days after the initial sampling event (Set 1). After the end of each sampling event, the heater tank reached the initial water temperature setpoint of 120 °F (≈49 °C) in approximately 40 min (Figure 1(b)). Although a portion of the hot water reached the target temperature of ≈49 °C, a water temperature gradient was measured inside the tank where a gradual decrease from ≈46 °C at the top of the tank to <35 °C at the bottom of the tank was observed (Figure 1(c)). This phenomenon is called thermal stratification, in which the fluid separates into regions of different temperatures with height. Water at the top of the tank generally maintained higher temperatures than water temperatures at the bottom. Previous studies identified design variables (e.g., side mounted heating elements, cold water inlet), mixing levels (e.g., stagnation), and insulation (e.g., heat loss) as the major contributor of thermal stratification of the water column in electric water heaters (Brazeau & Edwards 2013a; Yang *et al.* 2016; van Schalkwyk *et al.* 2022). However, when a recirculation pump is applied to a dedicated return line to the electric water heater, the stratification is disrupted by heat exchange between the layers inside the tank (Brazeau & Edwards 2013b). These systems are most often found in large occupational buildings such as hospitals, hotels, and offices, but have found their way into more homes. In contrast, the tank design (without recirculation pump) used in this study likely plays an important key role in thermal stratification, as a sharp decrease in temperature below the lower heating element and close to the internal cold water inlet was observed (Figure 1(c)). This area is located 20–25 cm from the base of the tank and occupies a volume of approximately 25 L. The temperature at this location ranged from 42 °C to a minimum of 35 °C, a temperature range that is favorable for the growth of most waterborne pathogens (Cabral 2010) including *L. pneumophila* (Katz & Hammel 1987). Environmental strains of *L. pneumophila* exhibited a notable capability for growth at lower temperatures than clinical strains (Sharaby *et al.* 2017).

### 3.2. The microbiome of the electric water heater

This study generated and analyzed 11 and 8 16S rRNA metagenome libraries from Sets 1 and 2, respectively (Table 1). Results from both sets of sequential sampling show that the microbial community is highly diverse. A total of 395 OTUs comprised of 142 bacterial genera were identified using the mothur software with the database GTDB release 207 (rarefied to 17,500 reads per sample). Taxonomic classification revealed that most of the diversity was associated with the classes Actinomycetia (47.21%), Alphaproteobacteria (23.28%), Gammaproteobacteria (13.48%), Vampirovibrionia (11.30%), Bacteroidia (1.32%), Acidobacteriae (1.06%), Nitrospiria (0.53%), Bdellovibrionia (0.45%), Planctomycetia (0.40%), Gemmatimonadetes (0.37%), Blastocatellia (0.24%), and Bacilli (0.11%) with additional representatives of 16 classes detected to a lesser extent (≤0.1% each). Similar taxonomic results were obtained on samples collected from bulk water and biofilms in 2021 by Gomez-Alvarez *et al.* (2023), which clearly identifies these taxa as major inhabitants of the HPS simulator. Further, 16S rRNA-based alpha diversity estimated an average richness of 103 (SD = 23, range = 60–158) and an observed Shannon diversity of 2.40 (SD = 0.37, range = 1.46–2.87) along the vertical stratification. The microbial distribution and growth in the water heater is directly associated with the complexity and design of the device, along with the variation in physicochemical parameters (Brazeau & Edwards 2013a).

### 3.3. Temperature stratification in water and their effects in the microbial ecology

**Figure 2 |:**
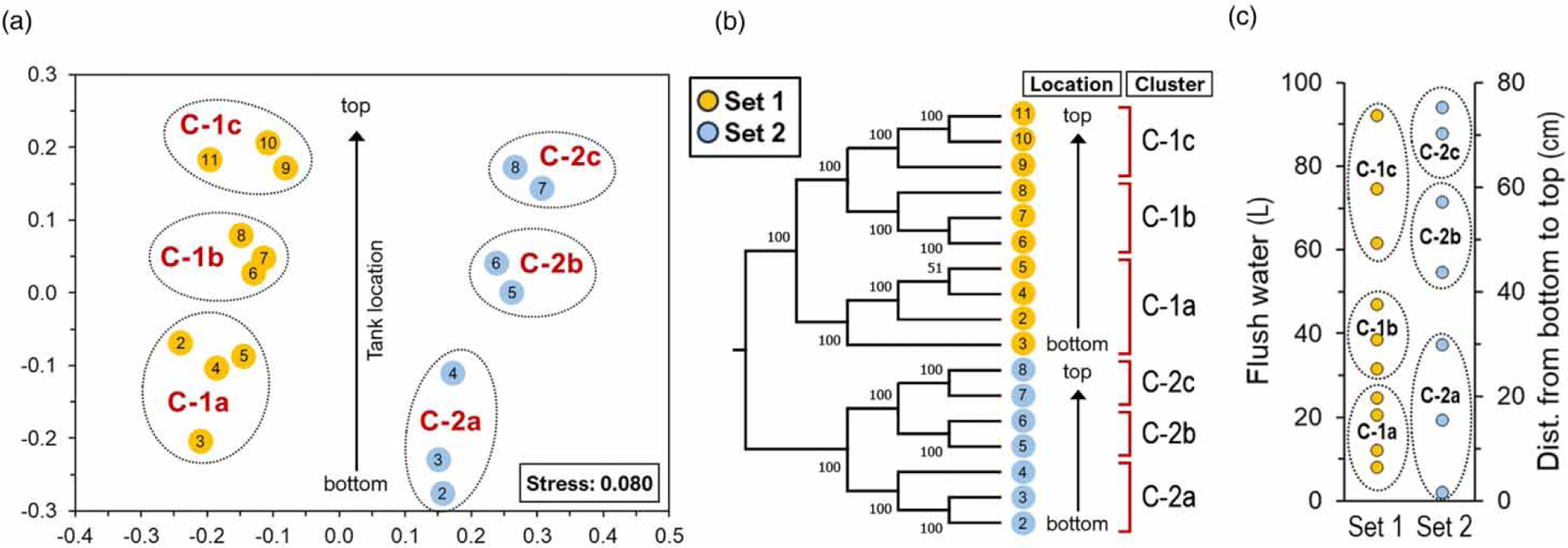
(a) Nonmetric multidimensional scaling (nMDS) ordination plot and (b) cluster analysis (CA) indicates depth-differentiation of microbial assemblages in a tank water heater. (c) Amount of flushed water (L) and locations (bottom to the top in cm) of stratified water samples in their respective microbial clusters (C-1 and C-2) in the water tank. Arrows indicate the orientation of samples in the water tank. Analyses were based on Jensen–Shannon dissimilarity of 16S rRNA OTU-level bacterial profiles (cutoff ¼ 0.03). Nodes in CA with a bootstrap value of .50% are indicated in the tree. OTU representatives that explained the dissimilarity within cluster of communities are listed in Figure 3.

The impact of thermal stratification on the microbial community in the water heater is evident (Figure 2). Temperature stratification in aquatic ecosystems creates vertical isolation of portions of the water body with the effect of stratification or clustering of the microbiome and their functional potential (i.e., biogeochemical pathways) through the water column (Yu *et al.* 2014; Zhang *et al.* 2015; Qu *et al.* 2018; Yue *et al.* 2021). In built environments such as drinking water storage tanks and water heaters, thermal stratification leads to the vertical compartmentalization of water temperature and nutrients, and also causes sedimentation. Currently, thermal stratification on the microbial community in built environments is poorly understood. Evidence from the current study indicated that the relative abundance of the classes Actinomycetia and Alpha-proteobacteria increased from the top to the bottom, while the classes Gammaproteobacteria and Vampirovibrionia showed the opposite trend in the vertical water column (Supplementary Figure S2). However, despite the variations in the community structure (i.e., abundance), the observed diversity and richness metrics remain consistent along the vertical stratification (Supplementary Figure S3, Mann–Whitney *U* test: *p >* 0.05). The composition (i.e., membership) of the microbiome remains almost identical, but their distribution varies along the vertical stratification. Brazeau & Edwards (2013a) documented the intricate relationships between temperature, disinfectant decay, and nutrients, and considered implications of stratification for microbial growth in water heater tanks. However, the scope of the study did not allow for the direct impact on the actual microbial community and their pathogen levels.

The nMDS (Figure 2(a)) and CA (Figure 2(b)) formed three defined clusters (Figure 2(c)) of vertical stratification in the water heater (PERMANOVA: *F* = 6.75, *p* = 0.0001). The nMDS stress score value of 0.08 indicated a good fit of the regression for the observed distances among the samples (Clarke 1993). The difference is explained by the small number of genus-level taxa (14 out of 142, representing ,10% of the observed genus) and explains 90% (SIMPER analysis) of the dissimilarity within water sections (i.e., clusters). The relative abundance of the 14 genus-level taxa varied significantly along the vertical stratification (Supplementary Table S2). Taxonomic analysis identified the taxa as members of the genera *Mycobacterium*, *Rubrivivax*, *Obscuribacter*, QKMZ01, *Erythrobacter*, VFBF01, *Methylobacterium*, *Sphingomonas*, *Sediminibacterium*, *Hyphomicrobium*, *Reyranella*, Ga0077553, *Lysobacter*, and *Blastococcus*. The 14 taxa are among the most abundant genus and represent 72% of the total distribution of reads in the metagenomic libraries. The rest of the taxa represent the rare biosphere and explain <10% of the dissimilarity suggesting a minor contribution to the differences found within the community. Nevertheless, the importance of the rare biosphere and its contribution to the overall function of the ecosystem must be considered (Gomez-Alvarez *et al.* 2016).

**Figure 3 |:**
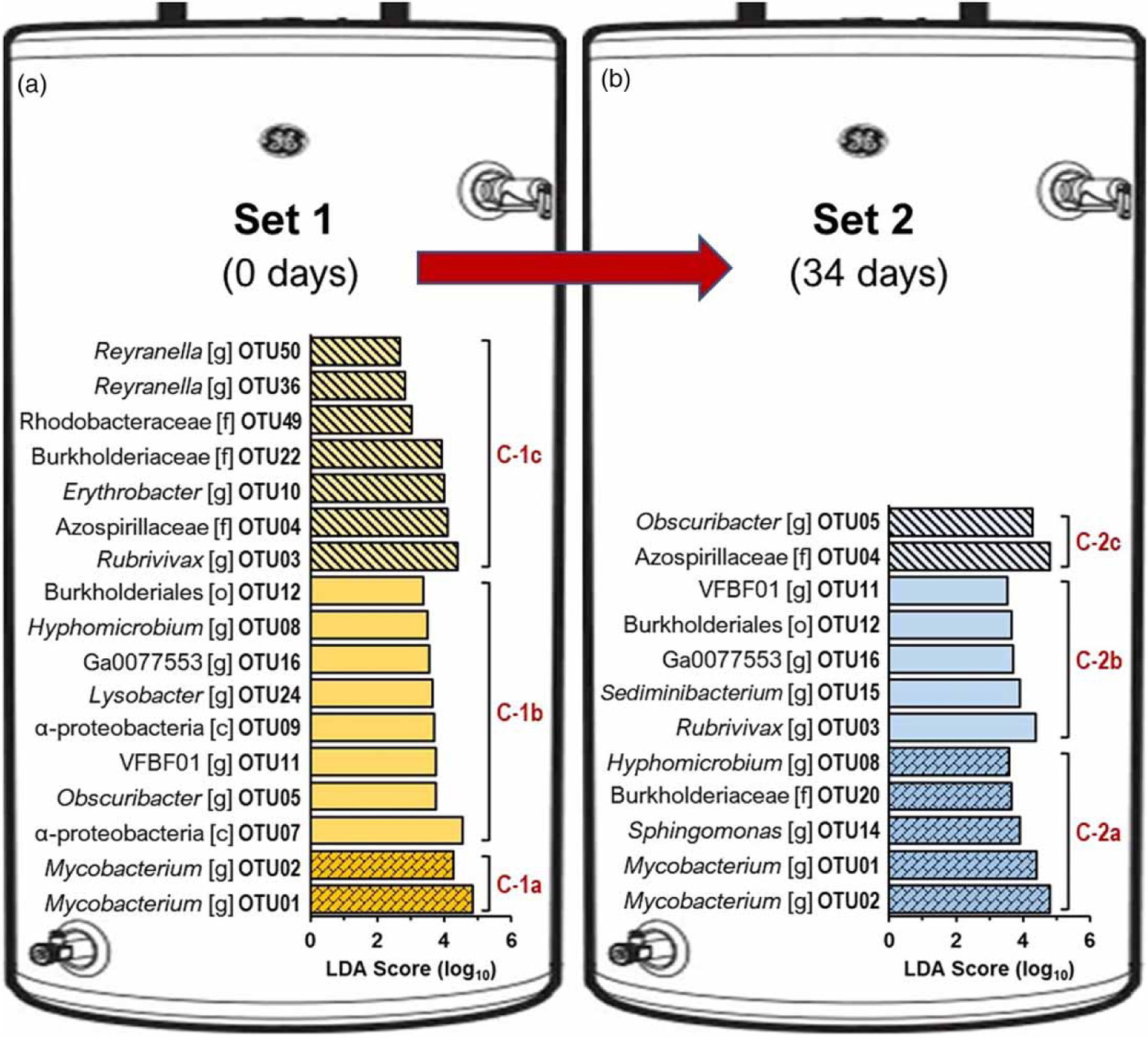
Identification of discriminative bacteria OTU-level indicators using linear discriminative analysis (LDA) effect size (LEfSe) analyses. The distribution histogram shows the bacterial OTU with LDA scores >4.0 that exhibit significant differences ( *p <*0.05) among clusters within (a) Set 1 and (b) Set 2. The length of the histogram represents the influence of the OTU. The letters c, o, f, and g indicate taxa-level assigned class, order, family, and genus, respectively.

The *Mycobacterium* genus represents 47% of the total diversity and has been frequently found in residential premise plumbing (Dowdell *et al.* 2019). Furthermore, representatives of this genus have emerged as a significant cause of opportunistic infections causing pulmonary disease with an increasing number of incidents associated with these infections (Ratnatunga *et al.* 2020). LEfSe analysis with an LDA threshold score of 2.0 identified two OTUs (OTU01 and OTU02) affiliated with the genus *Mycobacterium* overrepresented in the bottom section (Figure 3). The middle section was dominated by OTUs representing the genera *Rubrivivax* (OTU003), *Obscuribacter* (OTU005), *Hyphomicrobium* (OTU008), VFBF01 (OTU011), *Sediminibacterium* (OTU015), Ga0077553 (OTU016), and *Lysobacter* (OTU024). The dominant OTUs in the top section were closely related to members of the genera *Rubrivivax* (OTU003), *Obscuribacter* (OTU005), *Erythrobacter* (OTU010), *Reyranella* (OTU036 and OTU050), and the families Azospirillaceae (OTU004), Burkholderiaceae (OTU022), and Rhodobacteraceae (OTU049). The taxa identified in the heater tank are common inhabitants of drinking water systems (Abkar *et al.* 2024) and were previously identified in the HPS simulator (Gomez-Alvarez *et al.* 2023).

Overall, the microbiome composition remains almost identical, but their distribution varies along the vertical stratification. These findings are similar to those reported in previous studies of water stratification, albeit not at the same ecosystem scale (Cheng *et al.* 2020; Pavlovska *et al.* 2021; Yue *et al.* 2021). The results suggest that thermal stratification affects the community structure and to a smaller extent the microbial community composition. In many instances, such differences are largely controlled by deterministic factors and considered the prominent role of environmental filtering in influencing the assembly of the microbiome. Environmental filtering emphasizes the shaping of the community structure by abiotic factors (e.g., pH, temperature, and nutrients) (Kraft *et al.* 2015). But it is important to consider the complexity of this constrained ecosystem (i.e., heater tank), and therefore, we cannot discard unobserved ecological drivers including the constant interactions among microorganisms affecting the diversity (Krause *et al.* 2014; Aguilar-Trigueros *et al.* 2017). It is essential to reiterate that exploratory analyses may help reveal interesting patterns in data sets. The interpretation and explanation of the observations ultimately rely on the knowledge of the ecological situation of the ecosystem (Ramette 2007). More caution is needed in interpreting patterns and shifts in species abundance in communities along stratifications, as compelling evidence for environmental filtering (Kraft *et al.* 2015).

### 3.4. Partial drainage of water tank causes ecological disturbance

**Figure 4 |:**
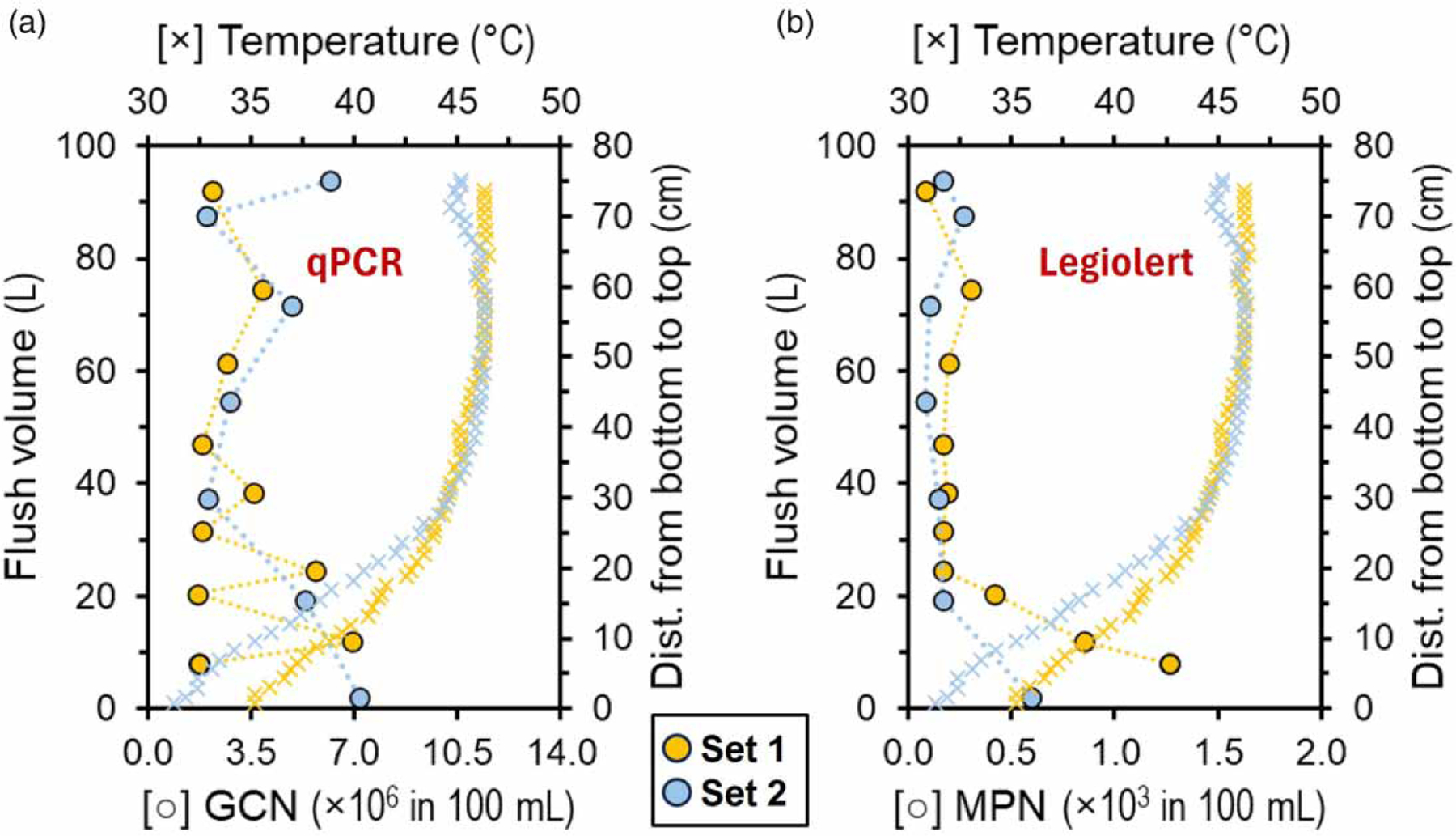
Cell density of *L. pneumophila* by qPCR (average GCN/100 mL) (a) and Legiolert method count (MPN/100 mL) (b) and relationship with temperature across stratified water samples. GCN, gene copy number; MPN, most probable number.

There is a common notion that episodes of disturbances induce a selection pressure on microbial populations (Allison & Martiny 2008). This work provides evidence that partial drainage of the water heater produces an effect in the microbial community structure (i.e., distribution of taxa). This effect was evident 34 days after partial drainage of the tank (Figure 2, PERMANOVA: *F* = 9.13, *p* = 0.0003). This disturbance can affect the stability of the microbial community directly as well as indirectly. SIMPER analysis identified the group of genus-level taxa responsible for the dissimilarity within water sections (Supplementary Table S2; see Section 3.3). The relative abundance of genus-level taxa and respective OTUs changes between Sets 1 and 2, but their composition does not. This may suggest that while the community is sensitive and does change, it is also resilient and can recover to its pre-disturbance condition (Shade *et al.* 2012). There is a growing consensus that microbial resilience is influenced by traits such as high abundances, widespread dispersal, and the potential for rapid growth rates (Allison & Martiny 2008). We hypothesized that the community is sensitive to disturbance and does change but is also resilient and quickly recovers to its initial composition, including the OPPPs. We observed that partial drainage of the tank does not produce short-term effects in thermal stratification and microbial community composition (i.e., membership). Further, it appears that partial drainage does not influence the prevalence and abundance for most of the targeted species in the water heater (Figure 4; Supplementary Figure S4). In most cases, the cell density remained consistent at previous levels. As observed previously with water management changes (i.e., disturbances), no long-term effect was observed from residential activity on cell density for most of the targeted species in the HPS simulator (Gomez-Alvarez *et al.* 2023). Partial drainage of the water tank demonstrated the resilience of the microbial community residing in this built environment with no significant mitigation of selected OPPPs (Mann–Whitney *U* test: *p >* 0.05). This study demonstrates that these organisms can survive and protect themselves from stressors during a disturbance event and respond by returning to their stable state after a brief shift in cell abundance (i.e., resilience). Dormancy, speciation rates, gene transfer, and dispersal abilities were identified as distinctive features of microbes intrinsically linked to microbial resilience (Philippot *et al.* 2021).

### 3.5. Electric heater tank provides favorable conditions for growth of pathogens

The growth of bacterial pathogens in premise plumbing may be unavoidable; however, understanding the locations and favorable conditions for growth in water heaters is important for ensuring public health. First, this study demonstrated that targeted OPPP and FLA populations, although sensitive to changes due to operational parameters (i.e., disturbance), maintained comparable cell density (Set 1 vs. Set 2) after partial drainage of the tank (Supplementary Figure S4). Previous studies have noted that these responses are determined by the location or type of environment in the HPS (Gomez-Alvarez *et al.* 2023). Indeed, the unique characteristics of heater tanks provide a favorable environment for the microbial establishment of OPPPs within the premise plumbing (Song *et al.* 2023). Previous studies identified design variables, mixing levels, and insulation as the major contributors of the microbial proliferation in electric water heaters (Brazeau & Edwards 2013a; Yang *et al.* 2016; van Schalkwyk *et al.* 2022).

Our observation showed that the cell density of *Mycobacterium* increased significantly at the bottom of the tank compared with the top section (Supplementary Figure S4, Mann–Whitney *U* test: *p* = 0.045). Further, results indicated that culturable *L. pneumophila* was positively correlated with a decrease in temperature at the bottom of the tank (Figure 4(b); Pearson, *r* = 0.68, *p* = 0.003). The temperature at the bottom decreased to 35 °C, which provides a favorable condition for the growth of *L. pneumophila* (Katz & Hammel 1987). In addition, the bottom of the tank is characterized by a lower chlorine residual (Gomez-Alvarez *et al.* 2023) with prolonged periods of stagnation and is considered the preferred niche for bacterial pathogens. Consequently, these favorable conditions found at the bottom of the electric tank promoted the establishment and further interaction of OPPPs and may be an important source of human exposure in residential and building structures. It is worth noting that qPCR consistently yielded higher values of *L. pneumophila* than culture methods (e.g., Legiolert), likely reflecting that qPCR measures the presence of all genetic material (i.e., from culturable, viable but nonculturable (VBNC), and dead cells, including free DNA) while culture methods only measure viable cells (Sylvestre *et al.* 2024). The discrepancies between culture methods and molecular assays highlight the necessity for adopting a standard method for *L. pneumophila* detection in water samples (Whiley & Taylor 2014). Overall, our study corroborated that electric hot water storage tanks are breeding grounds for microbes, including OPPPs. A previous study indicated the number of bacterial genera identified in the entry point (i.e., cold water intake) shared with the hot water heater is ,161, while the number of genera shared between the heater and point-of-use devices increased to 1,362, an 8.5-fold increase in taxa (Gomez-Alvarez *et al.* 2023). As a result, these devices can amplify the potential public health risk within the potable water system.

## 4. CONCLUSIONS

- The distribution of microbial communities showed evidence of vertical stratification with significant differences among different water depths.
- Vertical distribution of the microbial communities is greatly influenced by design variables (e.g., dual-side mounted heating elements) and sedimentation.
- The microbial community is highly diverse with temporal structuring influenced by the partial drainage (60% of the tank capacity) of the hot water tank.
- Partial drainage of the water tank demonstrated the resilience of the microbial community residing in this complex ecosystem with no significant mitigation of the microbiome and selected OPPPs.
- Environmental conditions and higher concentration of *L. pneumophila* at the bottom of the water tank suggest the potential growth and protection of opportunistic pathogen at this location.

## Supplementary Material

Supplementary Material 1

Supplementary Material 2

## Figures and Tables

**Table 1 |:** Water temperature, flushed volume, and distance of samples collected from the water tank

Sample	Temperature (°C) [bottom to top]	Volume (L) [flush +collected]	Distance^a^ (cm) [bottom to top]
Set 1			
T01	35.2	1.0 [0 + 1.0]	0.8
T02	37.3	8.0 [6.0 + 1.0]	6.4
T03	38.8	12.0 [3.0 + 1.0]	9.6
T04	41.4	20.5 [7.5 + 1.0]	16.4
T05	42.8	24.5 [3.0 + 1.0]	19.6
T06	43.9	31.5 [6.0 + 1.0]	25.2
T07	44.6	38.5 [6.0 + 1.0]	30.8
T08	45.2	47.0 [7.5 + 1.0]	37.6
T09	46.2	61.5 [13.5 + 1.0]	49.2
T10	46.2	74.5 [12.0 + 1.0]	59.6
T11	46.3	92.0 [16.5 + 1.0]	73.6
Set 2			
T01	31.3	1.0 [0 + 1.0]	0.8
T02	31.9	2.0 [0 + 1.0]	1.6
T03	38.3	19.4 [16.4 + 1.0]	15.5
T04	44.6	37.3 [16.9 + 1.0]	29.9
T05	46.1	54.5 [16.2 + 1.0]	43.6
T06	46.3	71.5 [16 + 1.0]	57.2
T07	45.1	87.7 [15.2 + 1.0]	70.2
T08	45.2	94. 0 [5.3 + 1.0]	75.2

a Distance measured from the bottom to the top of the water tank.

## Data Availability

All relevant data are included in the paper or its Supplementary Information.

## HIGHLIGHTS

- Microbial community response to a partial tank drainage.
- Evidence of vertical stratification in an electric heater tank.
- Community is sensitive to disturbance and does change but is also resilient.
- Growth and protection of opportunistic pathogens at the bottom of the tank.
