# Supplementary material for: Vertical stratification of the water microbiome in an electric water heater tank: implications for premise plumbing opportunistic pathogens
Source: J Water Health. Author manuscript; Available in PMC 2025 Mar 5. (PMC11880899; doi:10.2166/wh.2024.265)
Supplement: Supplementary Material 1 [file NIHMS2054967-supplement-Supplementary_Material_1.docx]

**Supplementary Material**

**Vertical Stratification of the Water Microbiome in an Electric Water Heater Tank: implications for premise plumbing opportunistic pathogens**

Vicente Gomez-Alvarez^*^, Hodon Ryu, Christy Muhlen, Daniel Williams, Darren Lytle, and Laura Boczek

U.S. Environmental Protection Agency, Office of Research and Development, Cincinnati, Ohio 45268

**Table of Contents**

|  |  | **Page** |
| --- | --- | --- |
| **Supplementary Materials and Methods** | | S2 |
| **Tables** |  |  |
| Table S1 | Primers and probes for qPCR assays | S6 |
| Table S1 | Distribution and taxonomy of genus-level representatives | S7 |
| **Figures** |  |  |
| Figure S1 | Diagram of home plumbing system (HPS) simulator | S8 |
| Figure S2 | Microbial community composition | S9 |
| Figure S3 | Bacterial richness and community diversity | S10 |
| Figure S4 | Cell density by qPCR analysis of targeted species | S11 |
| **References** |  | S12 |

**MATERIALS and METHODS**

*Home Plumbing System (HPS) Simulator*

The HPS simulator was constructed on January 23, 2012 (Lytle et al., 2021) and contained approximately 56 m Type M copper pipes (inside diameter 1.45 cm), a flow meter totalizer (located at the start of the simulator and recorded the flow rate of the whole simulator), an electric water heater, dishwasher, washing machine, a shower head, and four faucets. The water tank, shower head, toilet, and four faucets are connected via copper piping, brass fixtures, and solder joints. Faucet 1 is a cast brass utility faucet. Faucets 2 to 4 are three identical bathroom-type hot-and-cold water faucets with a chrome exterior and a cast brass and plastic interior. The HPS system was designed with a hot and cold water pipe system. The initial HPS design included a hot water recirculating pump, but the system was reverted to a traditional non-recirculating system in 2020. The lengths of the cold water line (in meters) from the flow meter to Faucet 1, Faucet 2, Faucet 3, Faucet 4, and the shower were 16.6, 14.8, 12.0, 9.1, and 15.5, respectively. The lengths of the hot water line (in meters) from the water heater to Faucet 1, Faucet 2, Faucet 3, Faucet 4, and the shower were 18.4, 16.1, 13.2, 10.2, and 16.4, respectively. Additional HPS simulator components included brass ball valves, brass check valves, bathtub, and a toilet.

The HPS was operated under a new “Random Reduced Normal Use” flushing protocol to simulate the average daily water use for a typical household of four residents (target total daily water use 70-80 gallons [≈265-303 L]). The faucets were given specific usage-type designations: three-bathroom taps (faucet 1, 2, and 4) and a kitchen tap (faucet 3). A dishwasher and washing machine were added to the HPS system. Household activities such as filling a glass of water, flushing the toilet, showering, and washing dishes were mimicked. The cold water lines and the hot water tank are fed with water that is supplied by the building water supply (Greater Cincinnati Water Works tap water); a water chemistry of pH 7.7 ± 0.3, alkalinity 76 ± 14 mg CaCO_3_/L, dissolved inorganic carbon 19 ± 4 mg C/L, calcium 28 ± 9 mg Ca/L, magnesium 8.4 ± 3.1 mg Mg/L, nitrate 0.88 ± 0.22 mg N/L, total phosphate 0.72 ± 0.57 mg PO_4_/L, and sulfate 58 ± 22 mg SO_4_/L during the study duration. The water heater generated hot water with an initial temperature set at 120°F (≈49°C). The target water use was achieved by manually flushing the hot and cold water for all faucets including the shower several times per weekday in a random schedule to simulate a typical home usage. The dishwashers and washing machines are run once a week. The toilet is flushed on a random schedule daily.

*DNA Extraction and Sequencing*

DNA of each water sample (approximately 1 L) was immediately extracted via filtration using 0.2 µm sterile membrane (Pall Corporation, Port Washington, NY) according to the manufacturer's protocol (DNeasy PowerWater kit, Qiagen). DNA concentration was measured using a NanoDrop ND-1000 UV spectrophotometer (NanoDrop Technologies, Wilmington, DE). DNA extracts were stored at -20°C until further processing. The V4 region of the 16S rRNA sequence was amplified using the bacterial primer set 515F and 806R (Caporaso et al., 2012). Paired-end 250 bp libraries were prepared using the Illumina MiSeq® Reagent v2 (500-cycles) kit on the MiSeq platform (Illumina Inc., San Diego, CA).

*16S rRNA sequence analysis*

Reads were analyzed using the software mothur v1.48.0 (Schloss et al., 2009) and were screened following the procedure described in Gomez-Alvarez et al. (2016). Briefly, fastq files with forward and reverse reads were used to form contigs. Reads were screened and removed if they (i) had a length less than 250 bp, (ii) contained ambiguous bases (N's), (iii) contained homopolymers greater than 7 bases, (iv) were identified as chimera, or (v) were classified as unknown, Chloroplasts, or Mitochondria. Reads were aligned against the SILVA SEED release 123 reference dataset and grouped with 97% sequence identity as the cut-off point for each Operational Taxonomic Unit (OTU). Prior to community analysis, samples were rarefied to the smallest dataset (17,500 reads).

*Taxonomic classification*

Taxonomic classification was obtained using the SBDI Sativa curated 16S GTDB r207 reference database (Parks et al., 2020). Sequences and taxonomic outline for the GTDB hierarchies were downloaded (SBDI 2021) and formatted for the purpose of using with the software mothur.

*Quantitative polymerase chain reaction (qPCR) analyses*

Culture-independent qPCR analyses were conducted for *Legionella* spp. and *Mycobacterium* spp. along with the free-living amoeba (FLA) *Vermamoeba vermiformis*. In addition, qPCR was used to monitor the occurrence of two major OPPPs: *Legionella pneumophila* and the nontuberculous mycobacterial *Mycobacterium intracellulare* (Table S1). A SYBR green qPCR assay was performed for *V. vermiformis* as described by Ryu et al., 2013. The assay was performed in a 25-μL reaction mixtures containing 1× Power SYBR green master mix (Applied Biosystems, Foster City, CA), 0.4 µM of each primer (final concentration), and 2 µL of the template. The amplification protocol involved an initial incubation at 50°C for 2 min, followed by 95°C for 10 min and 40 cycles of 95°C for 15 s and an annealing temperature of 56°C for 1 min (Table S1). Taqman qPCR assays were performed in a 25-μL reaction mixture containing 1× TaqMan universal PCR master mix (Applied Biosystems, Foster City, CA, USA), 0.4 µM of each primer (final concentration) and 0.2 µM of a 6-FAM (6-carboxyfluorescein)-labeled hydrolysis probe (final concentration), and 2 µL of the template. The amplification protocol involved 40 cycles of 95°C for 15 s and optimum annealing temperature for 1 min (Table S1).

Reaction mixtures were prepared in MicroAmp Optical 96-well reaction plates with MicroAmp Optical Caps (Applied Biosystems, Foster City, CA). All qPCR assays were performed using a QuantStudio^TM^ 6 Flex system (Applied Biosystems, Foster City, CA), followed by a melting curve analysis (i.e., from the annealing temperature to 90°C in 0.1-degree increments). Disassociation curves from the melting curve analysis were examined to determine the presence of potential primer-dimers and other non-specific reaction products. Signal intensity values were recorded for those reactions showing one corresponding amplification peak within the disassociation curves.

Two independent standard curves for each qPCR assay were generated by plotting threshold cycle (CT) values against the number of target gene copies corresponding to six 10-fold dilutions in duplicate of gBlock standards (IDT, Coralville, IA, USA). The target gene copy numbers (*T*) were estimated by the following equation:

*T* = [*D*/(*PL* × 660)] × 6.022 × 10^23^

where *D* (g/µL) is gBlock concentration, and *PL* (bp) is gBlock length in base pairs (Ryu et al., 2013). Percent amplification efficiencies were calculated by the instrument manufacturer’s instructions (Applied Biosystems, Foster City, CA). Two non-template controls per PCR plate were used to check for cross-contamination.

*Culturable* *Legionella pneumophila*

The presence and quantification of culturable *L. pneumophila* was determined using the 10 mL potable water Legiolert assay as outlined by the manufacturer (IDEXX, Westbrook, Maine). Briefly, water samples were shaken, and 10 mL was immediately transferred to a sterile bottle containing 90 mL of Butterfield’s buffer (Weber Scientific, Hamilton, NJ). A blister pack of Legiolert was then aseptically added to the 100 mL solution and shaken until dissolved. The solution was transfered to a Legiolert tray and sealed using the Quanti-Tray plus sealer. Trays were incubated in an incubator for 7 days at 39°C with deionized water for humidity. After 7 days, wells were counted as positive if they were turbid and/or had a brown color change. Positive wells were used to calculate the most probable number (MPN) as provided by the manufacturer.

*Data availability*

The raw sequence reads have been submitted to the NCBI Sequence Read Archive (SRA) under the BioProject [PRJNA961987](https://www.ncbi.nlm.nih.gov/bioproject/%20PRJNA961987) with the following BioSample numbers: [SAMN39596420](https://www.ncbi.nlm.nih.gov/biosample/SAMN39596420), [SAMN39596421](https://www.ncbi.nlm.nih.gov/biosample/SAMN39596421), [SAMN39596422](https://www.ncbi.nlm.nih.gov/biosample/SAMN39596422), [SAMN39596423](https://www.ncbi.nlm.nih.gov/biosample/SAMN39596423), [SAMN39596424](https://www.ncbi.nlm.nih.gov/biosample/SAMN39596424), [SAMN39596425](https://www.ncbi.nlm.nih.gov/biosample/SAMN39596425), [SAMN39596426](https://www.ncbi.nlm.nih.gov/biosample/SAMN39596426), [SAMN39596427](https://www.ncbi.nlm.nih.gov/biosample/SAMN39596427), [SAMN39596428](https://www.ncbi.nlm.nih.gov/biosample/SAMN39596428), [SAMN39596429](https://www.ncbi.nlm.nih.gov/biosample/SAMN39596429), [SAMN39596430](https://www.ncbi.nlm.nih.gov/biosample/SAMN39596430), [SAMN39596431](https://www.ncbi.nlm.nih.gov/biosample/SAMN39596431), [SAMN39596432](https://www.ncbi.nlm.nih.gov/biosample/SAMN39596432), [SAMN39596433](https://www.ncbi.nlm.nih.gov/biosample/SAMN39596433), [SAMN39596434](https://www.ncbi.nlm.nih.gov/biosample/SAMN39596434), [SAMN39596435](https://www.ncbi.nlm.nih.gov/biosample/SAMN39596435), [SAMN39596436](https://www.ncbi.nlm.nih.gov/biosample/SAMN39596436), [SAMN39596437](https://www.ncbi.nlm.nih.gov/biosample/SAMN39596437), and [SAMN39596438](https://www.ncbi.nlm.nih.gov/biosample/SAMN39596438).

**TABLES**

**Table S1** The sequences of primers and probes of qPCR assays.

| **Target species** | **Primer sequence** (5’-3’) | **Amplicon size** (bp) | **Annealing temp** (^o^C) | **References** | **Target genes** |
| --- | --- | --- | --- | --- | --- |
| *Legionella* spp. | 1055F: ATGGCTGTCGTCAGCT  1392R: ACGGGCGGTGTGTAC  16STaq1115: FAM-CAACGAGCGCAACCC-TAMRA | 168 | 50 | Lu et al., 2015 | 16S rRNA |
|  |  |  |  |  |  |
| *L. pneumophila* | Lpneu F1: CGGAATTACTGGGCGTAAAGG  Lpneu R1: GAGTCAACCAGTATTATCTGACCG  Lpneu P1: FAM-AAGCCCAGGAATTTCACAGAT-TAMRA | 100 | 60 | Donohue et al, 2014, 2019 | 16S rRNA |
|  |  |  |  |  |  |
| *Mycobacterium* spp. | 23SmycoF: GGGGTGTGGTGTTTGAG  23SmycoR: CTCCCACGTCCTTCATC  23SmycoProbe: FAM-TGGATAGTGGTTGCGAGCATC-TAMRA | 207 | 55 | Bruijnesteijn van Coppenraet et al., 2004 | ITS and 23S rRNA |
|  |  |  |  |  |  |
| *M. intracellulare* | MA/MI-F: GGGTGAGTAACACGTGTGCAA  MI-R: CCACCTAAAGACATGCGACTAAA  MA/MI-P: FAM-TGCACTTCGGGATAAGCCTGGGAAA-TAMRA | 100 | 60 | Chern et al., 2015 | 16S rRNA |
|  |  |  |  |  |  |
| *V. vermiformis* | Hv1227F: TTACGAGGTCAGGACACTGT  Hv1728R: GACCATCCGGAGTTCTCG | 502 | 56 | Kuiper et al., 2006; Lu et al., 2015 | 18S rRNA |

FAM, 6-carboxyfluorescein, fluorescence reporter dye; TAMRA, 6-carboxytetramethylrhodamine, fluorescence quencher dye.

**Table S2.** Distribution (%) and taxonomic affiliation of *Bacteria* domain genus-level representatives that explained ≈90% (SIMPER analysis) of the dissimilarity within water tank communities.

| **Taxa**^§^ | |  |  | **Set 1** |  |  |  | **Set 2** |  |  |
| --- | --- | --- | --- | --- | --- | --- | --- | --- | --- | --- |
| **Genus** | **Class** |  | **C-1a** | **C-1b** | **C-1c** | **∆**^†^ | **C-2a** | **C-2b** | **C-2c** | **∆** |
| *Mycobacterium* | Actinomycetia |  | 67.30 | 48.30 | 41.60 | **↓** | 48.90 | 30.60 | 23.90 | **↓** |
| *Rubrivivax* | Gammaproteobacteria |  | 2.51 | 8.64 | 13.80 | **↑** | 0.88 | 0.60 | 0.13 | **↓** |
| *Obscuribacter* | Vampirovibrionia |  | 3.29 | 4.51 | 5.16 | **↑** | 5.26 | 10.50 | 14.10 | **↑** |
| QKMZ01 | Vampirovibrionia |  | 1.85 | 2.53 | 1.86 | **↕** | 6.38 | 9.05 | 7.54 | **↕** |
| *Erythrobacter* | Alphaproteobacteria |  | 1.21 | 2.07 | 3.99 | **↑** | 0.75 | 0.38 | 0.51 | **↕** |
| VFBF01 | Alphaproteobacteria |  | 1.59 | 1.33 | 0.14 | **↓** | 3.18 | 0.98 | 0.30 | **↓** |
| *Methylobacterium* | Alphaproteobacteria |  | 1.31 | 0.84 | 0.27 | **↓** | 1.45 | 2.08 | 0.63 | **↕** |
| *Sphingomonas* | Alphaproteobacteria |  | 0.61 | 0.42 | 0.65 | **↕** | 2.90 | 1.20 | 0.40 | **↓** |
| *Sediminibacterium* | Bacteroidia |  | 0.26 | 0.46 | 0.66 | **↑** | 1.11 | 2.49 | 1.99 | **↕** |
| *Hyphomicrobium* | Alphaproteobacteria |  | 1.92 | 1.18 | 0.69 | **↓** | 1.64 | 0.62 | 0.41 | **↓** |
| *Reyranella* | Alphaproteobacteria |  | 0.17 | 0.40 | 0.69 | **↑** | 1.77 | 1.56 | 1.53 | **↓** |
| Ga0077553 | Terriglobia |  | 0.31 | 0.95 | 0.79 | **↕** | 1.06 | 2.40 | 1.36 | **↕** |
| *Lysobacter* | Gammaproteobacteria |  | 0.10 | 1.01 | 1.55 | **↑** | 0.02 | 0.13 | 0.01 | **↕** |
| *Blastococcus* | Actinomycetia |  | 0.97 | 0.00 | 0.11 | **↕** | 0.02 | 0.48 | 0.20 | **↕** |

^§^For comparison, the communities in all samples were normalized to the smallest library (*n* = 17,500) and clustered by locations in the water tank (see Fig. 2).

^†^Changes (∆) in the genus frequencies in the water tank: increase from the bottom to the top (**↑**), decrease from the top to the bottom (**↓**), no linear change (**↕).**

**FIGURES**


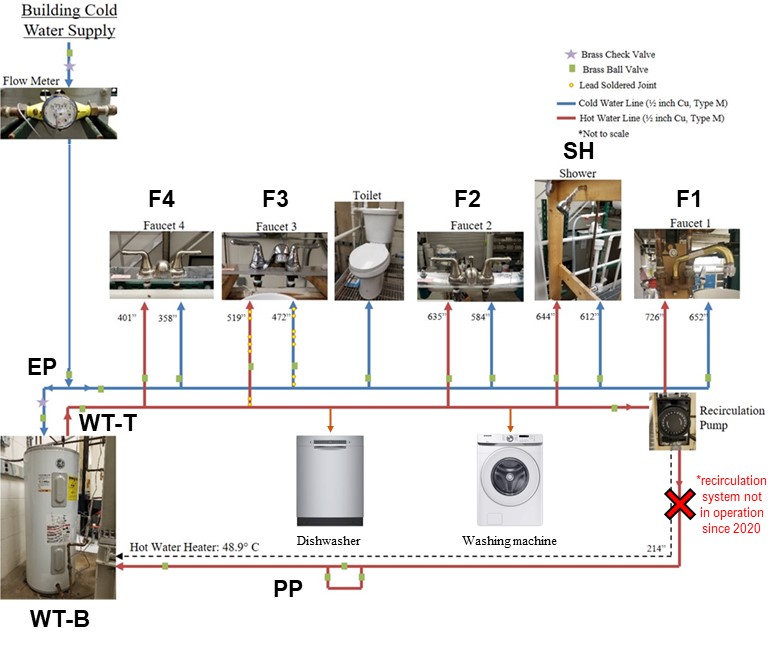


**Fig. S1.** Schematic of the HPS and sample port locations for hot (**▬**) and cold water (**▬**). Locations (sample ports): cold-water entry point (EP), hot water tank point of entry and return (WT-T and WT-B, respectively), premise plumbing (PP), faucets (F1, F2, F3, and F4), and the shower (SH). *HPS was reverted to a traditional non-recirculating system with the hot water recirculating system turned off and return pipe/valve shut-off in 2020.


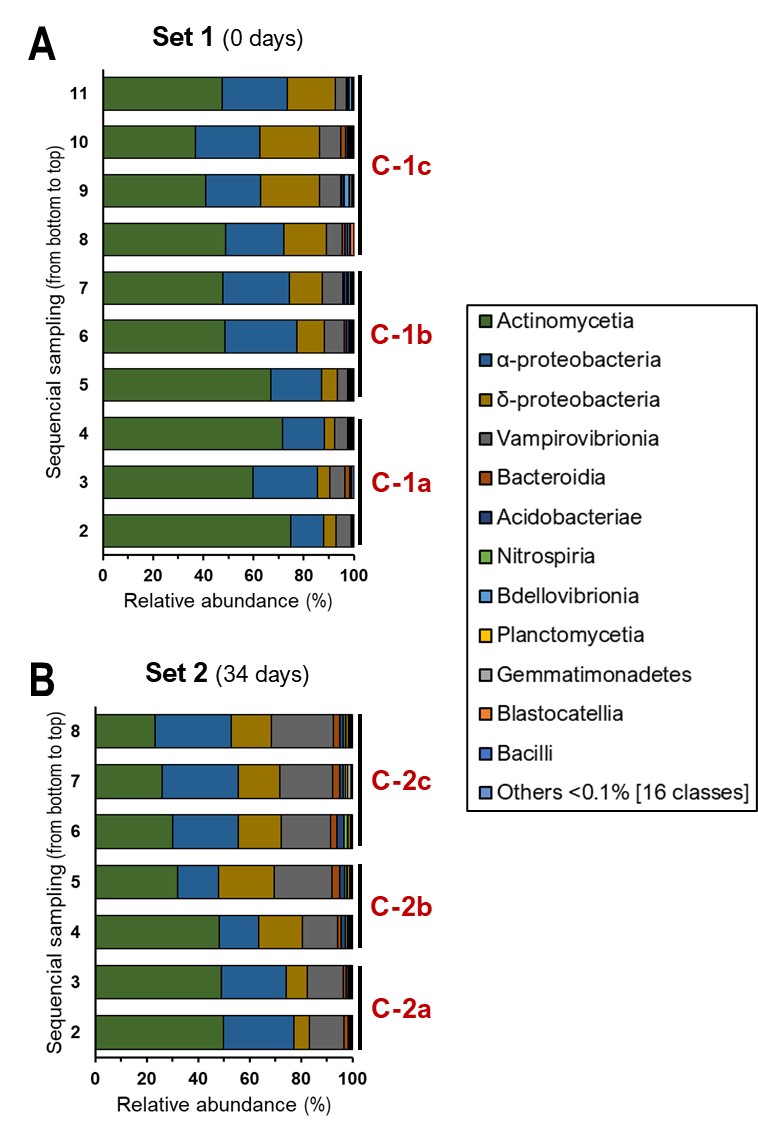


**Fig.S2.** Microbial community composition of (A) Set 1 and (B) Set 2 at the class level across stratified water samples.


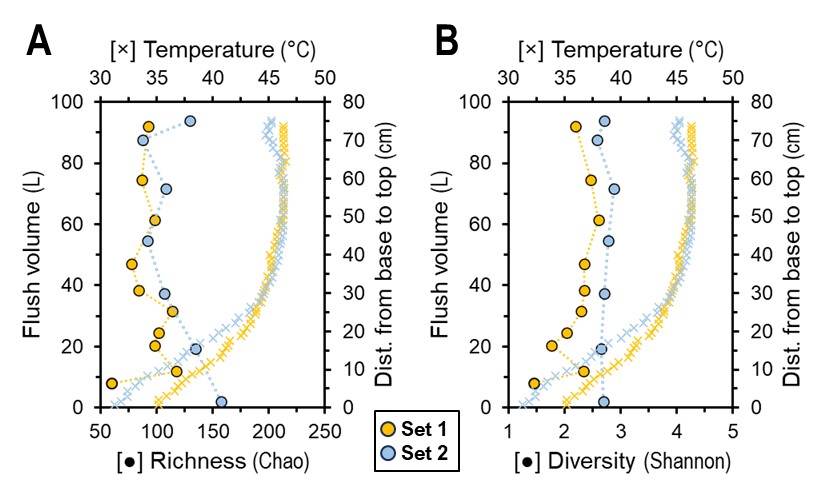


**Fig.S3.** (A) Community richness and (B) diversity estimates of the domain *Bacteria* and relationship with temperature across stratified water samples. For comparison, the read frequencies in all samples were normalized to the smallest library (*n* = 17,500). Richness is the total number of species in the community (i.e., the total number of independent sequences within the clone library; defined as aligned sequences with >97% similarity).


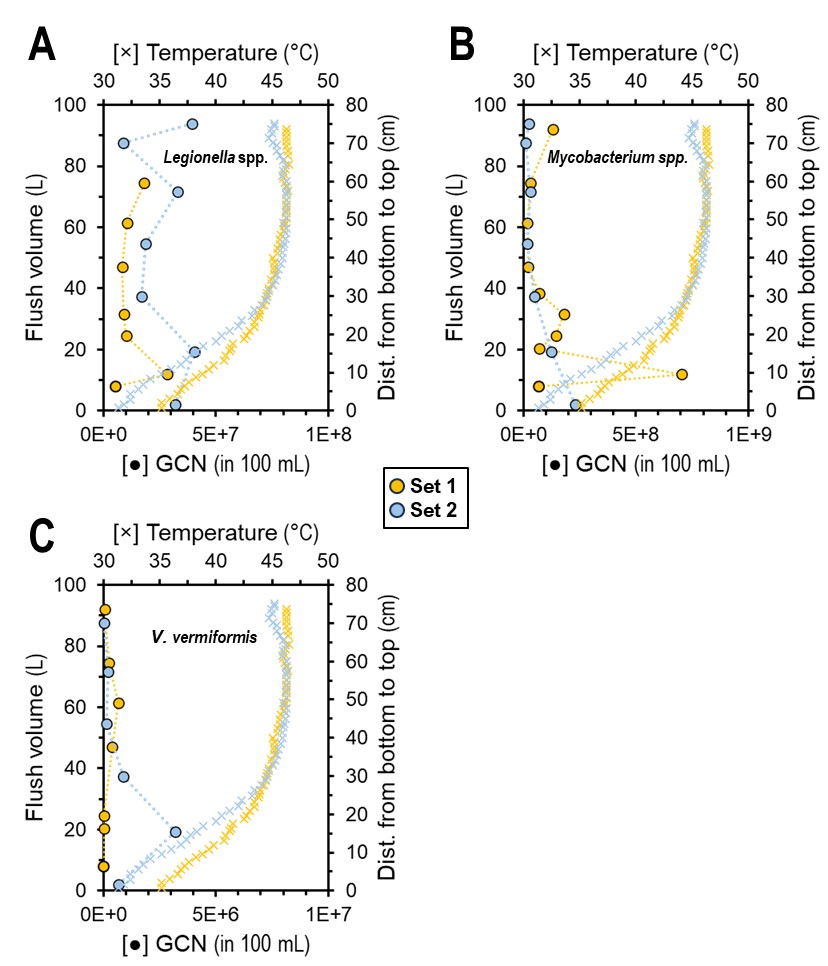


**Fig. S4.** Cell density by qPCR (average GCN/100 mL) of (A) *Legionella* spp., (B) *Mycobacterium* spp., and (C) and the free-living amoeba (FLA) specie *V. vermiformis* and relationship with temperature across stratified water samples. GCN: gene copy number.

**REFERENCES**

Bruijnesteijn Van Coppenraet, E. S., Lindeboom, J. A., Prins, J. M., Peeters, M. F., Claas, E. C., Kuijper, E. J. 2004 Real-time PCR assay using fine-needle aspirates and tissue biopsy specimens for rapid diagnosis of mycobacterial lymphadenitis in children. *J. Clin. Microbiol.* **42** (6), 2644-2650. https://doi.org/10.1128/JCM.42.6.2644-2650.2004

Caporaso, J. G., Lauber, C. L., Walters, W. A., Berg-Lyons, D., Huntley, J., Fierer, N., Owens, S.M., Betley, J., Fraser, L., Bauer, M., Gormley, N., Gilbert, J.A., Smith, G., Knight, R. 2012 Ultra-high-throughput microbial community analysis on the Illumina HiSeq and MiSeq platforms. *ISME J.* **6** (8), 1621-1624. https://doi.org/10.1038/ismej.2012.8.

Chern, E. C., King, D., Haugland, R., Pfaller, S. 2015 Evaluation of quantitative polymerase chain reaction assays targeting *Mycobacterium avium*, *M. intracellulare*, and *M. avium* subspecies *paratuberculosis* in drinking water biofilms. *J. Water Health* **13** (1), 131-139. https://doi.org/10.2166/wh.2014.060.

Donohue, M. J., O’Connell, K., Vesper, S. J., Mistry, J. H., King, D., Kostich, M., Pfaller, S. 2014 Widespread molecular detection of *Legionella pneumophila* serogroup 1 in cold water taps across the United States. *Environ. Sci. Technol.* **48** (6), 3145-3152. https://doi.org/10.1021/es4055115.

Donohue, M.J., Vesper, S., Mistry, J., Donohue, J. M. 2019 Impact of chlorine and chloramine on the detection and quantification of *Legionella pneumophila* and *Mycobacterium* species. *Appl. Environ. Microbiol.* **85** (24), e01942-19. https://doi.org/10.1128/AEM.01942-19.

Gomez-Alvarez, V., Pfaller, S., Pressman, J. G., Wahman, D. G., Revetta, R. P. 2016 Resilience of microbial communities in a simulated drinking water distribution system subjected to disturbances: role of conditionally rare taxa and potential implications for antibiotic-resistant bacteria. *Environ. Sci.: Water Res. Technol.* **2**, 645-657. https://doi.org/10.1039/C6EW00053C.

Kuiper, M. W., Valster, R. M., Wullings, B. A., Boonstra, H., Smidt, H., Van Der Kooij, D. 2006 Quantitative detection of the free-living amoeba *Hartmannella vermiformis* in surface water by using real-time PCR. *Appl. Environ. Microbiol.* **72** (9), 5750-5756. https://doi.org/10.1128/AEM.00085-06.

Lu, J., Struewing, I., Yelton, S., Ashbolt, N. 2015 Molecular survey of occurrence and quantity of *Legionella* spp., *Mycobacterium* spp., *Pseudomonas aeruginosa* and amoeba hosts in municipal drinking water storage tank sediments. *J. Appl. Microbiol.* **119** (1), 278-288. https://doi.org/10.1111/jam.12831.

Lytle, D.A., Formal, C., Cahalan, K., Muhlen, C., Triantafyllidou, S. 2021 The impact of sampling approach and daily water usage on lead levels measured at the tap. *Water Res.* **197**:117071. https://doi.org/10.1016/j.watres.2021.117071.

Parks, D.H., Chuvochina, M., Chaumeil, P. A., Rinke, C., Mussig, A. J., Hugenholtz, P. 2020 A complete domain-to-species taxonomy for Bacteria and Archaea. *Nat. Biotechnol.* **38** (9), 1079-1086. https://doi.org/10.1038/s41587-020-0501-8.

Ryu, H., Henson, M., Elk, M., Toledo-Hernandez, C., Griffith, J., Blackwood, D., Noble, R., Gourmelon, M., Glassmeyer, S., Santo Domingo, J. 2013 Development of quantitative PCR assays targeting 16S rRNA gene of *Enterococcus* spp. and their application to the identification of *Enterococcus* species in environmental samples. *Appl. Environ. Microbiol.* **79** (1), 196-204. https://doi.org/10.1128/AEM.02802-12.

Schloss, P. D., Westcott, S. L., Ryabin, T., Hall, J. R., Hartmann, M., Hollister, E. B., Lesniewski, R. A., Oakley, B. B., Parks, D.H., Robinson, C.J., Sahl, J. W., Stres, B., Thallinger, G. G., Van Horn, D. J., Weber, C. F. **2009** Introducing mothur: open-source, platform-independent, community-supported software for describing and comparing microbial communities. *Appl. Environ. Microbiol.* **75** (23), 7537-7541. https://doi.org/10.1128/AEM.01541-09.

Swedish Biodiversity Infrastructure (SBDI) 2021 SBDI Sativa curated 16S GTDB database. https://doi.org/10.17044/scilifelab.14869077.
